# Retraction: miR-203 Acts as a Tumor Suppressor Gene in Osteosarcoma by Regulating RAB22A

**DOI:** 10.1371/journal.pone.0282979

**Published:** 2023-03-29

**Authors:** The PLOS ONE Editors

The *PLOS ONE* Editors retract this article [1] because it was identified as one of a series of submissions for which we have concerns about peer review, authorship, and similarities across articles. Image similarities were noted between Fig 5C in [1] and Fig 3B in [2]. These concerns call into question the validity and provenance of the reported results. We regret that the issues were not addressed prior to the article’s publication.

All authors either did not respond directly or could not be reached.
